# m^6^A-modified circRNAs: detections, mechanisms, and prospects in cancers

**DOI:** 10.1186/s10020-022-00505-5

**Published:** 2022-07-14

**Authors:** Shiyi Qin, Qi Zhang, Yanhua Xu, Shuo Ma, Tianyi Wang, Yuejiao Huang, Shaoqing Ju

**Affiliations:** 1 Medical School of Nantong University, Nantong University, No. 19, Qixiu Road, Nantong, 226001 Jiangsu China; 2 Department of Laboratory Medicine, Affiliated Hospital of Nantong University, No. 20, Xisi Road, Nantong, 226001 Jiangsu China; 3 Department of Medical Oncology, Affiliated Tumor Hospital of Nantong University, Nantong, 226001 Jiangsu China; 4 Research Center of Clinical Medicine, Affiliated Hospital of Nantong University, Nantong, 226001 Jiangsu China

**Keywords:** CircRNA, Non-coding RNAs, N6-methyladenosine, Metabolism, Cancers

## Abstract

Circular RNAs (circRNAs) have become a research hotspot in recent years with their universality, diversity, stability, conservativeness, and spatiotemporal specificity. N^6^-methyladenosine (m^6^A), the most abundant modification in the eukaryotic cells, is engaged in the pathophysiological processes of various diseases. An increasing amount of evidence has suggested that m^6^A modification is common in circRNAs and is associated with their biological functions. This review summarizes the effects of m^6^A modification on circRNAs and their regulation mechanisms in cancers, providing some suggestions of m^6^A-modified circRNAs in cancer therapy.

## Background

Circular RNAs (CircRNAs) were first discovered in the 1970s and were initially used to represent splicing errors before serving as a by-product of splicing (Sanger et al. 1976). Subsequently, a large number of biologically significant circRNAs have merged and come to the attention of scholars. Abnormally expressed circRNAs are commonly linked to various human diseases such as cardiovascular diseases (CVDs), kidney diseases, immunity, and cancers (Gomes et al. 2020; Jan van Zonneveld et al. 2021; Chen et al. 2019a; Shang et al. 2019). Therefore, circRNAs hold great promise for cancer diagnosis and treatment thanks to their universality, diversity, stability, conservativeness, and spatiotemporal specificity (Kristensen et al. 2019).

More than 170 chemically distinct types of modifications have been identified in messenger RNAs (mRNAs) and a few non-coding RNAs (ncRNAs) of eukaryotes, bacteria and archaea, giving rise to RNA epigenetics (Boccaletto et al. 2022). The most popular RNA modifications include N^6^-methyladenosines (m^6^A), 5-methylcytosines (m^5^C), 5-hydroxymethylcytosine (5hmC), N^1^-methyladenosines (m^1^A), N^6^, 2′-Odimethyladenosine (m^6^Am), 7-methylguanine (m^7^G), and pseudouridine (Ψ) (Nombela et al. 2021). Among them, the m^6^A modification is the most abundant base modification in eukaryotic cells with a typical consensus sequence RRACH motif (R = G or A; H = A, C, or U) (Dominissini et al. 2012). Generally, those bases are enriched in the coding sequence (CDS), 3′-untranslated regions (3′-UTRs), and near stop codons of mRNAs (Meyer et al. 2012).

Recently, the m^6^A modification in the N^6^ position of adenosine has been found in circRNAs (Yang et al. 2017). However, the regulatory network between m^6^A modification and circRNAs remains complex. This review, centered on the roles of m^6^A modification on circRNAs, summarizes the existing detection methods and databases for m^6^A-modified circRNAs. The regulatory mechanisms of m^6^A-modified circRNAs in cancers and their effects on chemoradiotherapy resistance are reviewed to provide a comprehensive understanding of cancer diagnosis and treatment.

## Biogenesis, characteristics and biological functions of circRNAs

### Biogenesis of circRNAs

**Fig. 1:**
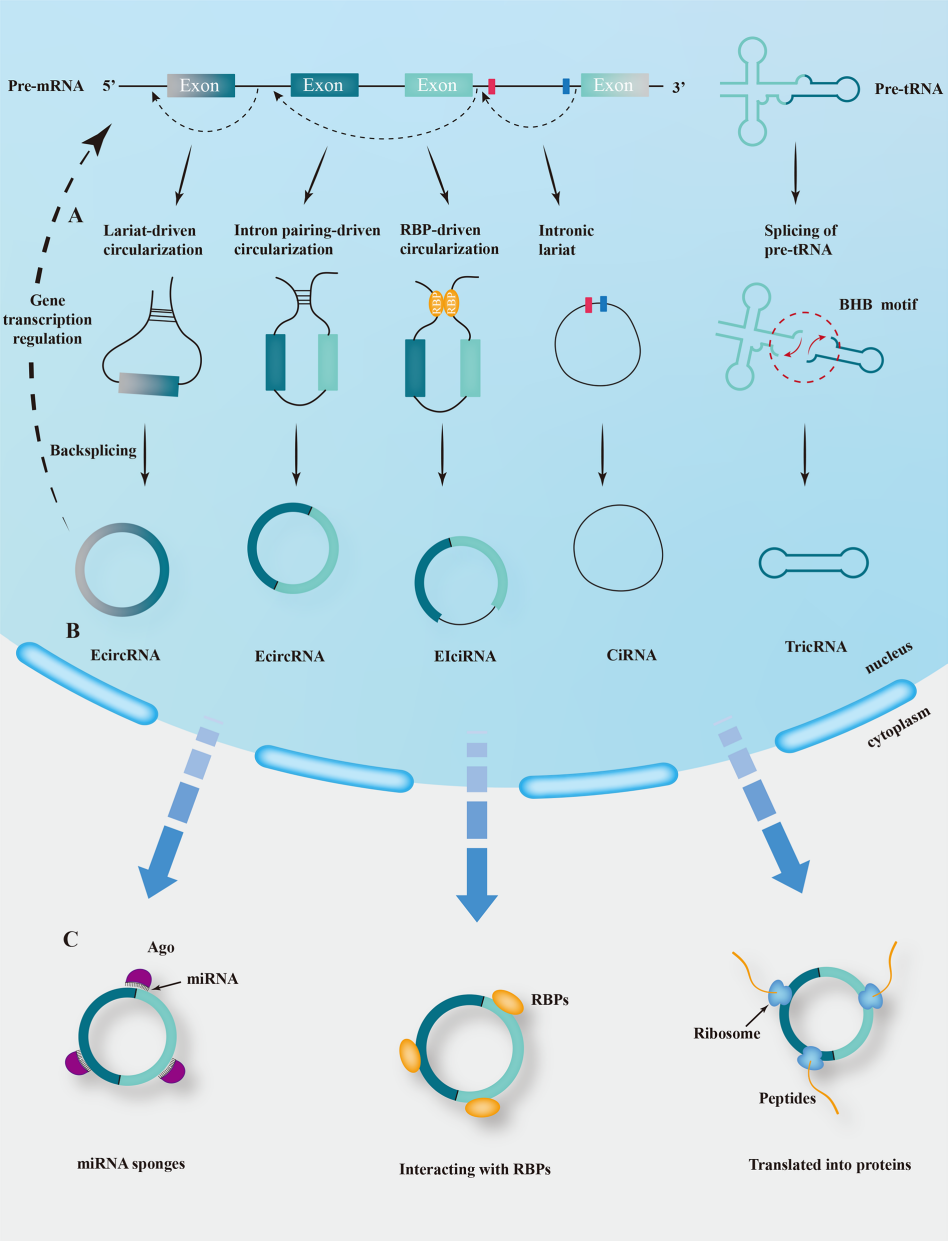
Biogenesis and biological functions of circRNAs. **A** The biogenesis models of circRNAs include lariat-driven circularization, intron pairing-driven circularization, RBP-driven circularization, intronic lariat, and splicing of pre-tRNA.** B** Based on the origin of circRNA, it can be divided into four categories, namely EcircRNA, EIciRNA, CiRNA, and TricRNA. **C** CircRNAs serve four main biological functions, including acting as miRNA sponges, interacting with RBPs, translating into proteins, and regulating gene transcription

CircRNAs have proliferated and are primarily generated by the back-splicing of pre-mRNAs. Four biogenesis models of circRNAs have been discovered, including lariat-driven circularization, intron pairing-driven circularization, RNA binding proteins (RBPs)-driven circularization, and intronic lariat (Kristensen et al. 2019). Besides, a small fraction of intron-derived circRNAs can also be generated by pre-tRNA. Briefly, the tRNA splicing nucleic acid endonuclease (TSEN) complex cleaves the intron-containing pre-tRNA at a typical bulge-helix-bulge (BHB) motif and then the resultant intron termini are joined by RtcB ligase to form a stable circRNA (Lu et al. 2015; Schmidt et al. 2019) (Fig. 1A). CircRNAs can thus be divided into four types based on their origins, including: exonic circRNAs (EcircRNAs), exon–intron circRNAs (EIciRNAs), intronic circRNAs (CiRNAs), and others, such as tRNA intronic circular RNAs (TricRNAs) (Schmidt et al. 2019; Zhang et al. 2013) (Fig. 1B).

### Characteristics of circRNAs

CircRNAs are found in nearly all mammals (Ji et al. 2019), plants (Wang et al. 2014), parasites (Broadbent et al. 2015), archaea (Danan et al. 2012), and viruses (Nahand et al. 2020). Particularly approximately 9% of expressed genes in human tissues can generate corresponding circRNAs in human heart, and 20% of genes can produce circRNAs in the brain (Aufiero et al. 2018; Rybak-Wolf et al. 2015). Researchers have validated more than 25,000 human fibroblast RNAs with backsplices as circRNAs (Jeck et al. 2013). Furthermore, the same genes can generate various types of circRNAs through alternative circularization (Salzman et al. 2012). Unlike linear RNAs with 5′ and 3′ ends, circRNAs have a covalently closed loop structure generated from primary transcripts by back-splicing (Jeck et al. 2013). CircRNAs are more stable than linear RNAs because the former ones do not have free ends, and therefore are resistant to foreign chemicals or exonuclease interference, and they have a long half-life of more than 48 h (Suzuki et al. 2006; Enuka et al. 2016). In this sense, circRNA can affect cell functions by accumulating in cells with slower division rates. CircRNAs are also highly conserved. One study has shown that approximately 20% of human circRNAs are homologous to mouse circRNAs (Guo et al. 2014). Another study discovered that approximately 20% of porcine splice sites involved in circRNA production are functionally conserved between mice and humans (Venø et al. 2015). Last but not least, circRNAs, which are dynamically expressed in a spatiotemporal manner, especially during mammalian brain development, have varied expression levels during the developmental process and at different regulation levels, making them more likely to be a disease biomarker (Venø et al. 2015; You et al. 2015).

### Biological functions of circRNAs

As research advances, circRNAs have received increased attention for their biological functions, as evidenced by the following aspects. (i) Being as microRNA (miRNA) sponges. Many circRNAs have specific binding sites to miRNAs that can reduce the activity of miRNAs while increasing that of miRNA target genes. CircRNAs, as competing endogenous RNAs (ceRNAs) remain the most classical mechanism of tumor regulation (Hansen et al. 2013). (ii) Interacting with RBPs. Some circRNAs contain specific protein binding sites that bind to RBPs and regulate target RNA, thus fostering the linear splicing of the gene and parental gene transcription (Ashwal-Fluss et al. 2014). (iii) Being translated into proteins. Some circRNAs have proven to be translated by the IRES-dependent mechanism, and ribosomes can be recruited by IRES-transacting factors (ITAFs) to initiate translation in the absence of typical translation initiation factors (Jiang et al. 2021; Xia et al. 2019). Besides, m^6^A-modified circRNAs can function in cap-independent translation, which will be discussed further below. (iv) Regulating gene transcription. Some researchers claim that some circRNAs in the nucleus can regulate gene transcription and thus perform specific physiological functions. For example, some CiRNAs and EIciRNAs, such as Ci-ankrd52, EIciPAIP2, and EIciEIF3J, are abundant in the nucleus and associated with RNA Pol II to promote transcription of their parental genes (Li et al. 2015). It is worth mentioning that circRNAs can also act as regulators affecting mRNA translation and stability (Wu et al. 2019a; Huang et al. 2020) (Fig. 1C). Therefore, circRNAs have wide range of biological functions that need further exploration.

## M^6^A writers, erasers, and readers

M^6^A modifications on circRNAs can be installed, removed, and recognized by the same m^6^A regulators in mRNAs, known as “writers” (methyltransferases), “erasers” (demethylases), and “readers” (recognitions).

### M^6^A writers/methyltransferases

Generally, m^6^A modification are installed by various methyltransferases acting on specific RNAs, but most of them are installed by the multicomponent m^6^A methyltransferases complex (MTC, also named “writers”), with methyltransferase-like 3 and 14 proteins (METTL3 and METTL14) as its core components (Wang et al. 2016). Other MTC components, such as Wilms Tumor 1 Associated Protein (WTAP) (Ping et al. 2014), Vir-like m^6^A methyltransferase associated (VIRMA, also called “Virilizer” or “KIAA1429”) (Schwartz et al. 2014), RNA recognition motif 15/15B (RBM15/15B) (Patil et al. 2016), Zinc finger CCCH domain-containing protein 13 (ZC3H13) (Knuckles et al. 2018), and Cbl proto-oncogene-like 1 (CBLL1, also known as “HAKAI”) (Bawankar et al. 2021), also play roles in facilitating the complex’s recruitment to specific sites and maintaining its stability. Aside from the enzymes mentioned above involved in MTC formation, methyltransferase-like 16 (METTL16) (Pendleton et al. 2017), methyltransferase-like 5 (METTL5) (Tran et al. 2019), and Zinc finger CCCH-Type containing 4 (ZCCHC4) (Ma et al. 2019) have been discovered to be independent RNA methyltransferases. However, these methyltransferases can only catalyze a few m^6^A residues in RNAs (Pendleton et al. 2017; Tran et al. 2019; Pinto et al. 2020).

### M^6^A erasers/demethylases

M^6^A methylation is a dynamic, multi-layered, and reversible process that can be removed by erasers (also known as “demethylases”). Fat mass and obesity-associated protein (FTO, also known as “ALKBH9”) and AlkB homolog 5 (ALKBH5) belong to the AlkB subfamily of Fe (II)/α-ketoglutaric acid (αKG) dioxygenase, and they can catalyze the demethylation of m^6^A in both αKG and Fe (II) dependence(Jia et al. 2011; Zheng et al. 2013).

### M^6^A readers/recognitions

**Fig. 2:**
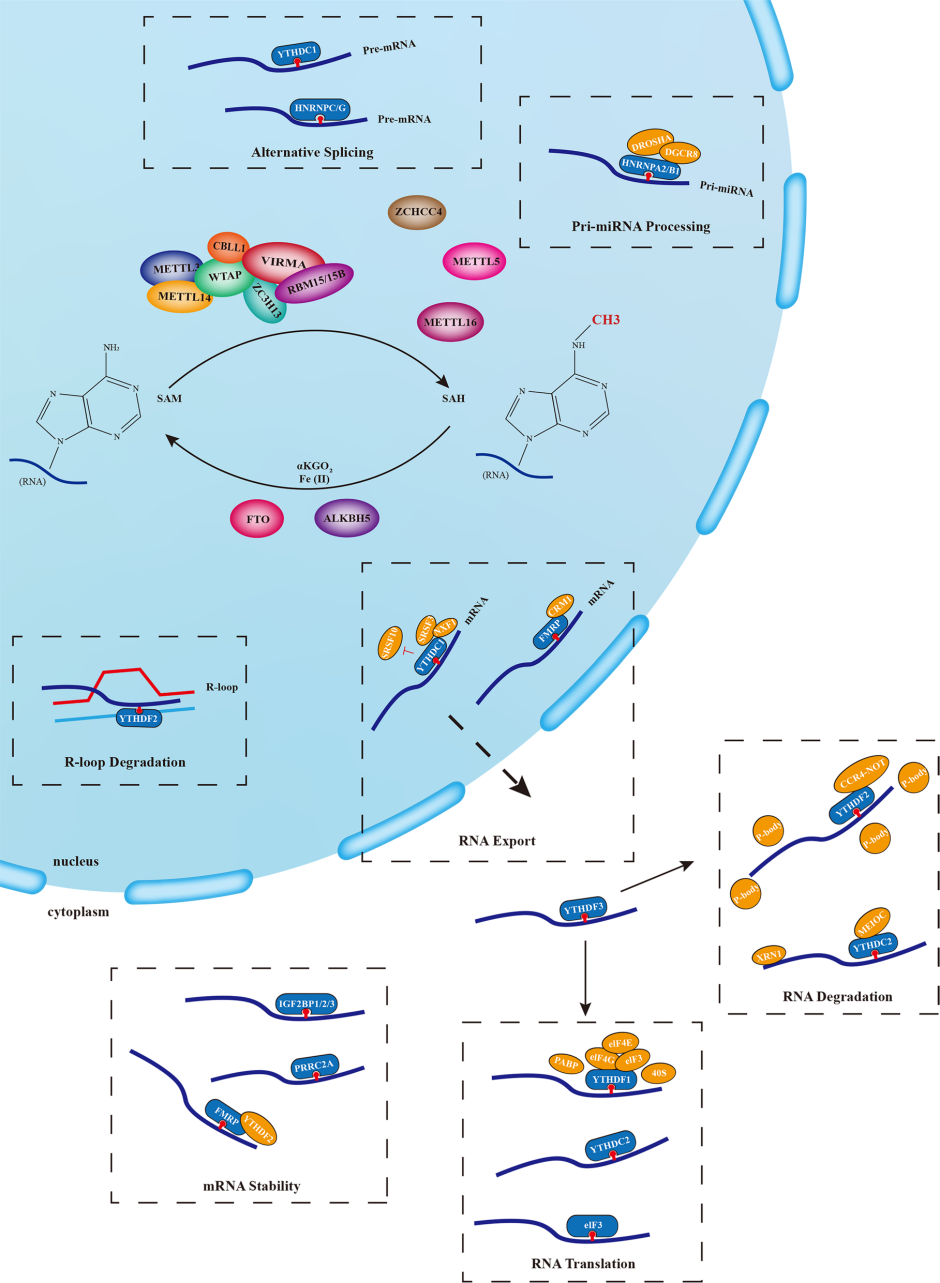
The dynamic and reversible process of m^6^A modification. The m^6^A modification can be installed by the multicomponent m^6^A methyltransferases complex (writers) which includes METTL3, METTL14, WTAP, VIRMA, RBM15/15B, ZC3H13 and CBLL1, as well as independent RNA methyltransferases such as METTL16, METTL5, ZCCHC4, and removed by demethylases (erasers) FTO and ALKBH5. Various binding proteins (readers) can then recognize the m^6^A modification to perform specific biological functions. In the nucleus, m^6^A can be identified by YTHDC1, HNRNPC/G, HNRNPA2B1, YTHDF2 and FMRP, and is involved in RNA alternative splicing, nuclear RNA processing, R-loop degradation, and RNA export. In the cytoplasm, m^6^A can be identified by YTHDF1/2/3, YTHDC2, IGF2BPs, eIF3, FMRP and PRRC2A, and regulates RNA stability, translation, and degradation

Numerous studies have revealed that m^6^A modifications can be recognized by various binding proteins (also called readers) to perform specific biological functions. To date, several readers have been extensively studied. Take YT521-B homology (YTH) domain family for example. It contains five proteins: YTH domain family protein 1 (YTHDF1), YTH domain family protein 2 (YTHDF2), YTH domain family protein 3 (YTHDF3), YTH domain containing 1 (YTHDC1), and YTH domain containing 2 (YTHDC2) (Liu et al. 2015). The first three are typically found in the cytoplasm to perform their functions. Among them, YTHDF2 can interact with the carbon catabolite repressor 4-negative on TATA (CCR4-NOT) complex to transport RNA to the processing body (P-body), thereby degrading RNA (Du et al. 2016). Besides, YTHDF1 and YTHDF3 have been found to act synergically to mediate m^6^A modifications in RNAs and affect the initiate translation of RNA with eukaryotic initiation factor 3, 4E, and 4G (eIF3, eIF4E, and eIF4G), poly(A) binding protein (PABP), and the 40S ribosomal subunit in a cap-dependent manner (Wang et al. 2015; Shi et al. 2017). However, a recent study has found that YTHDF2 can also exist in the nucleus, interact with m^6^A modifications on RNA within R-loops, and destabilize the RNA: DNA hybrids, thus regulating the accumulation of R-loops, and playing a role in safeguarding genomic stability (Abakir et al. 2020). YTHDC1 is also nuclear enriched and primarily involved in the selective splicing and nuclear transport of m^6^A transcripts (Widagdo et al. 2022). YTHDC2, which occurs in the cytoplasm and plays a vital role in RNA decay via interactions with adaptor proteins, and in RNA translation efficiency (Wojtas et al. 2017; Mao et al. 2019). In addition to the YTH domain family, heterogeneous nuclear ribonucleoprotein C1/C2 (HNRNPC), heterogeneous nuclear ribonucleoprotein G (HNRNPG), and heterogeneous nuclear ribonucleoprotein A2B1 (HNRNPA2B1), as part of the heterogeneous nuclear ribonucleoprotein (HNRNP) family are involved in alternative splicing and nuclear RNA processing (Alarcón et al. 2015; Liu et al. 2017). Furthermore, it has been proposed that eIF3 initiates translation in a cap-independent manner by binding to the m^6^A sites in the 5′-UTR of mRNAs (Meyer et al. 2015), while insulin-like growth factor 2 mRNA-binding protein 1/2/3 (IGF2BP1/2/3) can enhance the stability and translation of the target RNAs in the cytoplasm (Zhang et al. 2018; Wu et al. 2019b). Similar to IGF2BP1/2/3, fragile X mental retardation protein (FMRP) and proline-rich spiral coil 2A (PRRC2A) can also maintain the stability of their target RNAs. Furthermore, it is worth noting that FMRP can also occur in the nucleus and take part in the nuclear export of m^6^A-enriched RNAs (Hsu et al. 2019) (Fig. 2).

In summary, those m^6^A regulators, particularly readers, are complex and diverse. Their effects on m^6^A-modified circRNAs in cancers are discussed in detail below.

## Detection methods and databases for m^6^A-modified circRNAs

Over the past decades, further research into the functions of m^6^A-mediated circRNAs has been limited by a lack of suitable detection methods and databases. However, with the continuous improvement of multiple detection methods and databases, especially the emergence of the next-generation sequencing (NGS), the field of m^6^A methylation has seen a dramatic shift.

### Quantitative and semi-quantitative detection of m^6^A-modified circRNAs

As a semi-quantitative method for determining the level of overall m^6^A-modified circRNAs, dot blot is easy to operate and time-saving, but it only can confirm the presence of m^6^A or compare the amount of m^6^A in different groups, but cannot quantify or locate m^6^A (Zhou et al. 2017). In addition, the m^6^A level detection is a colorimetric method for quantifying the overall level of m^6^A RNA methylation in total RNAs, mRNAs, and ncRNAs. The concept of the test is similar to enzyme-linked immunosorbent assay (ELISA) and easy to operate (Ge et al. 2020). However, Ribonuclease R (RNase R) should be first used to de-linearize for quantifying the overall m^6^A level in total circRNAs and more research papers will be required to validate the method in the future. Besides, Methylated RNA immunoprecipitation (MeRIP) assay (m^6^A RIP), is a method for enriching m^6^A-modified circRNAs by using an anti-m^6^A antibody and quantitative real-time polymerase chain reaction (qPCR) to identify the enriched circRNAs. This method is convenient and only requires a kit to perform an experiment, but it lacks specificity (Chen et al. 2021). Moreover, the m^6^A-circRNA epitranscriptomic microarray in combination with a dual-color fluorescence microarray labeling system and RNA modification immunoprecipitation, allows for the quantitative detection of the percentage of epigenetic modifications in each transcript with a low total RNA requirement and high specificity. This method, however, is not widely used and deserves more attention (Fan et al. 2022) (Table 1).

**Table 1:** Detection methods for m^6^A-modified circRNAs

Methods	References
Quantitative and semi-quantitative detection	
Dot blot	Zhou et al. (2017)
M^6^A level detection	Ge et al. (2020)
MeRIP assay/m^6^A RIP	Chen et al. (2021)
M^6^A-circRNA epitranscriptomic microarray	Fan et al. (2022)
The detection of m^6^A modification sites	
MeRIP-seq/m^6^A-seq	Dominissini et al. (2012), Antanaviciute et al. (2017)
MazF PCR	Imanishi et al. (2017)
T3 DNA ligase-dependent PCR	Liu et al. (2018)
Nanopore DRS	Zhao et al. (2019)

### The detection of m^6^A modification sites in circRNAs

Although most relevant methods focus on detecting m^6^A modifications in linear RNAs, the precise detection of m^6^A modification sites in circRNAs remains uncommon. Methylated RNA immunoprecipitation and sequencing (MeRIP-seq/m^6^A-seq), is a predominant method for detecting m^6^A modifications in RNAs. It mainly combines anti-m^6^A antibody with m^6^A-containing RNA fragments for NGS. The m^6^A-seq approach has some limitations: (i) It can only identify m^6^A hypermethylation enrichment regions on RNAs with a resolution of about 100nt, but cannot locate individual m^6^A sites; (ii) It requires a large number of total RNA samples due to its low sensitivity; (iii) Antibodies to m^6^A can recognize modifications similar to m^6^A, such as m^6^Am, with less specificity (Dominissini et al. 2012; Antanaviciute et al. 2017). Notably, a variety of antibody-independent methods for detecting m^6^A modifications have been discovered in recent years. For example, MazF PCR is a single-base m^6^A detection method that uses the m^6^A-sensitive RNA endonuclease MazF, which has been found to cleave RNAs with non-methylated ACA sequence, but not those with the methylated m6ACA sequence. However, to cover all the RRACH motifs in the transcriptome, new enzymes that recognize more universal sequence motifs must be explored (Imanishi et al. 2017). Besides, the T3 DNA ligase-dependent PCR assay is a highly sensitive and selective single-base detection that can locate m^6^A modification fraction at any specific RNA site. It is worth noting that both MazF PCR and ligase-dependent PCR assays for detecting m^6^A sites in circRNAs require RNase R to digest linear RNA before performing such validations (Liu et al. 2018). Furthermore, nanopore-based direct RNA sequencing (nanopore DRS) is another single-base detection method that locate m^6^A modifications in circRNAs by enriching circRNAs in samples, fragmenting and sequencing them on nanopore platforms, with high efficiency and simplicity (Wang et al. 2020). However, more studies are needed to validate the application of the aforementioned antibody-independent methods in m^6^A-related fundamental studies and clinical diagnosis (Table 1).

### Databases for predicting m^6^A-modified circRNAs

The databases for predicting m^6^A methylation sites of circRNAs include Ensembl (Howe et al. 2021), Circm6A (Ye et al. 2021), TransCirc (Huang et al. 2021), SRAMP (Zhou et al. 2016), RMVar (Luo et al. 2021), RMBase V2.0 (Xuan et al. 2018), circBank (Liu et al. 2019), and DeepM6ASeq (Zhang and Hamada 2018). These databases can predict not only m^6^A modifications but also circRNAs with miRNA binding sites, protein-coding potential, conservations, mutations, etc. Notably, m6A2Target is a novel comprehensive database for exploring the target genes of writers, erasers, and readers of m^6^A modification (Deng et al. 2021). Thanks to their convenience, simplicity, and data visualization, those databases facilitate scientific research (Table 2).

**Table 2:** Databases for predicting m^6^A-modified circRNAs

Name	Website	Characteristics	Reference
Ensembl	http://rapid.ensembl.org	It is a genome browser can be used to identify m^6^A modification sites with the RRACH motif	Howe et al. (2021)
Circm6A	https://github.com/canceromics/circm6a	It is a powerful tool for detecting m^6^A modification of circRNA	Ye et al. (2021)
TransCirc	https://www.biosino.org/transcirc/	It is a database that mainly predict translatable circRNA and circRNA m^6^A modification sites	Huang et al. (2021)
SRAMP	http://www.cuilab.cn/sramp	It can extract and integrate the sequence and predict structural features around m^6^A sites	Zhou et al. (2016)
RMVar	http://m6avar.renlab.org	It can be used to search for m^6^A-associated variants and diseases	Luo et al. (2021)
RMBase V2.0	http://rna.sysu.edu.cn/rmbase	It is a comprehensive database for exploring post-transcriptionally modifications of RNAs and their relationships with microRNA binding events, disease-related SNPs, and RBPs	Xuan et al. (2018)
circBank	www.circbank.cn	It is a comprehensive database for predicting circRNAs with miRNA binding sites, protein coding potential, conservations, mutations, and m^6^A modifications	Liu et al. 2019)
DeepM6ASeq	https://github.com/rreybeyb/DeepM6ASeq	It is a deep-learning-based framework to predict m^6^A-containing sequences and visualize saliency map for sequences	Zhang and Hamada (2018)
m6A2Target	http://m6a2target.canceromics.org/#/home	It is a comprehensive database for the target gene of writers, erasers and readers of m^6^A modification	Deng et al. (2021)

## Role of m^6^A modifications on circRNAs

### M^6^A modification mediates circRNAs translation

**Fig. 3:**
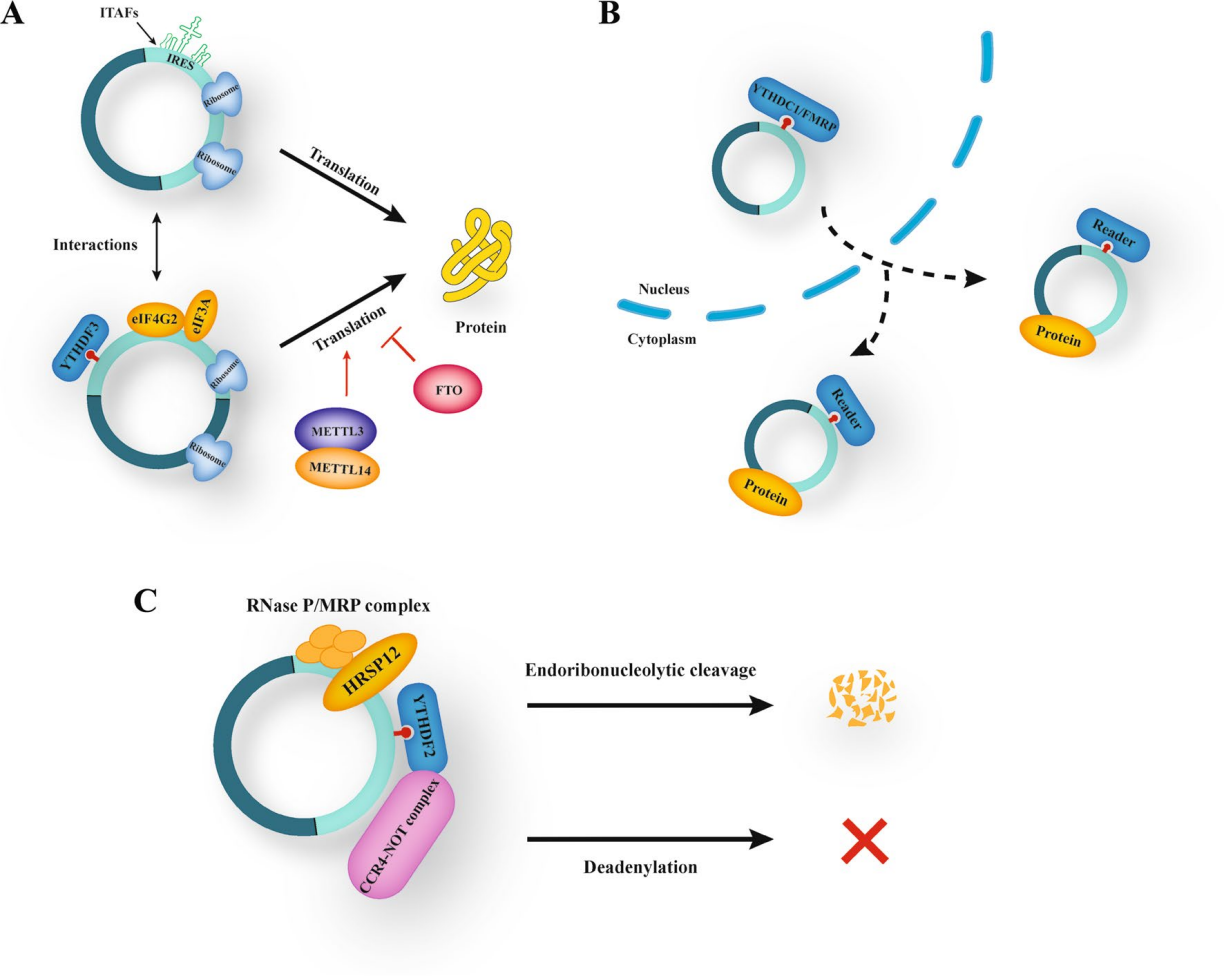
Role of M^6^A modifications on circRNAs. **A** M^6^A modification mediates circRNAs translation. M^6^A-driven translation of circRNAs relies on YTHDF3, eIF4G2 and eIF3A. Meanwhile, the process can be enhanced by METTL3/14 and inhibited by FTO. Besides, it is suggested that an interaction may exist between the IRES-driven translation and m^6^A-driven translation. **B** M^6^A modification mediates circRNAs nucleoplasmic transport. The m^6^A readers, such as YTHDC1 and FMRP, could induce the nuclear and cytoplasmic transport of circRNAs. **C** M^6^A modification regulates the stability of circRNAs. M^6^A-modified circRNAs can be endoribonuclease-cleaved via YTHDF2-HRSP12-RNase P/MRP axis

Accumulating evidence indicates that circRNAs code mainly through the IRES-driven translation and m^6^A-driven translation. Studies found that circRNAs containing m^6^A residues can be translated cap-independently. For example, Yang et al. discovered that the m^6^A-driven translation of circRNAs relies on the reading protein YTHDF3, as well as eukaryotic translation initiation factor 4 gamma 2 (eIF4G2) and eukaryotic initiation factor 3A (eIF3A) and that this process can be enhanced by methyltransferase METTL3/14 and inhibited by demethylase FTO. Moreover, further assays have indicated that an m^6^A site is sufficient to initiate translation and identify 33 peptides encoded by the back-splice junctions of m^6^A-modified circRNAs. These 33 peptides do not match any known proteins in the UniProt database but can be identified through proteomic analyses, suggesting that the m^6^A-driven translation of circRNAs widespread in the human transcriptome (Yang et al. 2017). Similarly, in human papillomavirus (HPV), circE7 with m^6^A modification can be translated into the E7 tumor protein (Zhao et al. 2019). Besides, studies have pointed out that m^6^A modifications can initiate and regulate circRNAs translation. Previous studies have discovered that circ-ZNF609 can be translated through the IRES-driven manner, while the latest one has identified that m^6^A-modified circ-ZNF609 can drive cap-independent translation through YTHDF3 and elF4G2. The above-mentioned findings suggest that the possibility of an interaction between the two forms that drive the translation of circRNAs. However, the specific correlation between them needs to be further explored (Legnini et al. 2017; Timoteo et al. 2020) (Fig. 3A).

To summarize, all those findings provide more possibilities for exploring the translation of m^6^A-driven circRNAs.

### M^6^A modification mediates nucleoplasmic transport of circRNAs

In recent years, many published articles have shown that individual circRNAs can be transported into the cytoplasm during biogenesis and development, competing with other RNAs for binding by RBPs or miRNAs (Memczak et al. 2013). Therefore, it is crucial to understand how circRNAs export from nucleus to the cytoplasm. In drosophila, researchers have found that the Drosophila DExH/D-box helicase at 25E (Hel25E) interference significantly enriches circRNAs in the nucleus. In human cells, circRNAs have been discovered to be transported from the nucleus to the cytoplasm in a transcript-length-dependent manner via drosophila Hel25E and its human homologs, ATP-dependent RNA helicase DDX39A (also termed as nuclear RNA helicase URH49) and spliceosomal RNA helicase DDX39B (also termed as dead box protein UAP56) (Huang et al. 2018). Besides, Chen et al. identified that circ1662 overexpression increases the nuclear yes-associated protein 1 (YAP1) and decreases the cytoplasmic YAP1, indicating that circ1662 could promote YAP1 nuclear transport. Further function assays have confirmed that circ1662 promotes colorectal cancer (CRC) invasion and migration by accelerating YAP1 nuclear transport (Chen et al. 2021). In addition, the m^6^A reader YTHDC1 can bind to circNSUN2 and facilitate circNSUN2 to export from the nucleus to the cytoplasm in an m^6^A-dependent manner, and to promote colorectal liver metastasis through the circNSUN2-IGF2BP2-High Mobility Group AT-Hook 2 (HMGA2) RNA–protein ternary complex in the cytoplasm (Chen et al. 2019b). Furthermore, YTHDC1 and FMRP have been identified as readers to recognize HBV transcripts with m^6^A methylation modification and facilitate their transport to the cytoplasm (Kim et al. 2021) (Fig. 3B).

Consequently, the m^6^A modification can affect the nuclear and cytoplasmic transport of circRNAs by interacting with proteins.

### M^6^A modification regulates the stability of circRNAs

RNase R and actinomycin D assays have shown that circRNAs are more stable than their origin genes, because they are not easily degraded by nucleic acid exonucleases and have a long half-life. Nonetheless, a recent study has pointed out that circRNAs can be degraded in some unique manners. For example, Hansen et al. revealed that the removal of the circular cerebellar degeneration associated protein 1 (CDR1) antisense transcripts with perfect complementary miRNA target sites could be mediated by miR-671 in an Argonaute2 (Ago2)-slicer-dependent manner. However, it does not work for circRNAs that lack miRNA sponge function or specific miRNA target sites (Hansen et al. 2011). Another study has reported that the depletion of GW182, a key component of P-body and RNA interference (RNAi) machine, can accumulate steady-state circRNA transcripts. However, that of other P-body components or RNAi machine factors does not affect circRNA levels, indicating that GW182 is a major factor in circRNA degradation. Nevertheless, the specific mechanisms remain to be further investigated (Jia et al. 2019). Aside from the above-mentioned findings, YTHDF2-heat-responsive protein 12 (HRSP12)-ribonuclease P (RNase P)/mitochondrial RNA processing (MRP) is the most common way of endoribonucleolytic cleavage of m^6^A-modified circRNAs. HRSP12 acts as an adapter protein that links YTHDF2 and RNase P/MRP, rapidly degrading YTHDF2-bound circRNAs (Park et al. 2019) (Fig. 3C).

Therefore, the complexities in the degradation of m^6^A circRNAs could contribute to the more dynamic regulation of m^6^A-modified circRNAs during various biological and physiological processes.

## M^6^A-modified circRNAs in cancers

### Colorectal cancer

Colorectal cancer (CRC) has been reported to rank third in incidence, and second in mortality according to the latest research. It accounts for about one in ten cancer cases and deaths (Sung et al. 2021). Therefore, specific mechanisms must be explored to better understand the CRC progression.

By using the MeRIP assay, Gene Expression Omnibus (GEO), and The Cancer Genome Atlas (TCGA) databases, researchers have found that circ3823 is enriched in the m^6^A precipitated fraction and have speculated that YTHDF3 and ALKBH5 cooperate with YTHDF2 to degrade circ3823, demonstrating that circ3823 might promote CRC growth, metastasis, and angiogenesis via circ3823/miR-30c-5p/Transcription factor 7 (TCF7) axis (Guo et al. 2021). Besides, refractory metastatic CRC is usually the leading cause of death in CRC patients (Hofheinz and Stintzing 2019). For example, Chen et al. demonstrated that METTL3 can induce circ1662 formation by installing m^6^A modifications in its flanking reverse complementary sequences via MeRIP assay, thus promoting epithelial-mesenchymal transition (EMT) and accelerating lung metastases of CRC via the YAP1-mothers against decapentaplegic homolog 3 (SMAD3) axis (Chen et al. 2021). Additionally, another study has identified that m^6^A-modified circNSUN2 is frequently upregulated in CRC patients with liver metastasis (LM), indicating a lower patient survival. MeRIP assay and other assays first verified that circNSUN2 is highly enriched in the m^6^A precipitated fraction and YTHDC1 can promote cytoplasmic export of m^6^A-modified circNSUN2. Further assays have indicated that circNSUN2 enhances the stability of HMGA2 mRNA by forming a circNSUN2/IGF2BP2/HMGA2 ternary complex in the cytoplasm, thus leading to the LM of CRC (Chen et al. 2019b).

In conclusion, those findings suggest that m^6^A-modified circRNAs may play a vital role in the CRC progression and serve as a potential diagnostic and therapeutic target for CRC, especially the metastasis-related CRC.

### Gastric cancer

In the latest global cancer report, gastric cancer (GC) is the fifth most common cancer and the fourth leading cause of cancer death worldwide (Sung et al. 2021). Therefore, further study of the molecular mechanism underlying GC is required.

Zhang et al. first predicted the potential m^6^A sites of the top 20 differentiated expressed circRNAs (DECs) by adopting the SRAMP database and m^6^A RIP assays, indicating that the m^6^A level of DECs is positively correlated with the DEC expression level in gastric tissues and may be closely related to circRNA functionality. Nevertheless, more research into the potential functions and mechanisms of m^6^A modification on identified DECs in poorly differentiated gastric adenocarcinoma (PDGA) is needed (Zhang et al. 2020). M^6^A-circRNA epitranscriptomic microarray and MeRIP assays have revealed that METTL14 can regulate the m^6^A level and expression of circORC5, and that METTL14-mediated circORC5 can sponge miR-30c-2-3p to regulate AKT1 substrate 1 (AKT1S1) and eukaryotic translation initiation factor 4B (EIF4B) expression in GC cells, thereby promoting GC progression (Fan et al. 2022).

Overall, those findings shed light on how m^6^A-modified circRNAs contribute to GC.

### Liver cancer

Liver cancer is the sixth most common cancer and the third leading cause of cancer death worldwide, among which hepatocellular carcinoma (HCC) comprises 75–85% (Sung et al. 2021). Several studies have shown that m^6^A-modified circRNAs are involved in HCC regulation.

In the study of Chi et al., circMAP2K4 was validated to promote HCC biogenesis via the miR-139-5p/YTHDF1 axis. Then, the expression and prognostic value of all m^6^A RNA methylation modulators and the biological pathways were evaluated by TCGA and International Cancer Genome Consortium (ICGC) databases, indicating that the circRNA regulatory network based on hsa-miR-139-5p/YTHDF1 axis is involved in regulating m^6^A RNA methylation modulators (Chi et al. 2021). Besides, Liu et al. observed that KIAA1429 is negatively correlated with m^6^A-modified circDLC1 after the intersection of RNA-seq and m^6^A-seq approaches. Further assays have found that circDLC1 binds to Human Antigen R (HuR) and blocks the interaction between HuR and matrix metalloproteinase 1 (MMP1) mRNAs, suggesting that m^6^A-regulated circDLC1 may serve as a therapeutic target for HCC (Liu et al. 2021). Additionally, MeRIP-seq, SRAMP database, and m^6^A RIP assays have confirmed that circHPS5 is highly m^6^A-modified, and METTL3 can mediate the circHPS5 formation. YTHDC1 can expedite the cytoplasmic output of m^6^A-modified circHPS5, making circHPS5 act as a miR-370 sponge to regulate HMGA2 expression and accelerate HCC cell development (Rong et al. 2021).

Hence, those findings convincingly indicated that m^6^A regulated-circRNAs may serve as potential therapeutic targets for liver cancer.

### Breast cancer

Breast cancer (BC) is the fifth leading cause of cancer mortality, surpassing lung cancer as the leading cause worldwide (Sung et al. 2021). Therefore, identifying novel mechanisms and therapeutic targets is crucial for BC treatment.

Fortunately, the circBank database and m^6^A RIP assays have revealed that circMETTL3 is highly enriched in m^6^A precipitated fraction, and its expression is affected by the m^6^A modification. CircMETTL3 can sponge miR-31-5p to upregulate cyclin-dependent kinases (CKD1) expression, thus promoting BC progression (Li et al. 2021a).

Those findings indicated that circMETTL3 may act as a potential therapeutic target for BC. Nevertheless, the role of m^6^A-modified circRNAs in BC is rarely reported and deserves more attention.

### Cervical cancer

Cervical cancer (CC) is the fourth most commonly diagnosed cancer and the fourth leading cause of cancer death in women (Sung et al. 2021).

M^6^A-RIP assay has confirmed that METTL3 can mediate the m^6^A modification level of human papillomavirus (HPV)-derived circE7. Further assays have revealed that circE7 can encode E7 oncoprotein in a heat-shock regulated manner and that the mutation of the potential m^6^A motifs of circE7 can strongly inhibit E7 oncoprotein expression, implying that m^6^A-modified circE7 plays a vital role in the translation mechanism (Zhao et al. 2019). Besides, another study has found that m^6^A-modified circARHGAP12 can interact with the m^6^A reader IGF2BP2 to enhance forkhead box M1 (FOXM1) mRNA stability and thus allow CC cells to proliferate and migrate(Ji et al. 2021).

In summary, those achievements might provide ideas for the targeted therapy based on the mechanisms of m^6^A-modified circRNAs regulating CC tumorigenesis.

### Lung cancer

Lung cancer remains the leading cause of cancer morbidity and mortality worldwide, with non-small cell lung cancer (NSCLC) accounting for about 80–85% (Sung et al. 2021). Despite recent advances in NSCLC treatment, the overall cure and survival rates remain low (Hirsch et al. 2017). Therefore, it is crucial to study and figure out the molecular mechanism of NSCLC to improve its prognosis.

In the study by Li et al., the MeRIP assay revealed that circNDUFB2 is considerably enriched in m^6^A modification, and that METTL3/14 plays a significant role in affecting the interactions between circNDUFB2 and IGF2BPs. CircNDUFB2 not only acts as a scaffold by forming a tripartite motif containing 25 (TRIM25)/circNDUFB2/IGF2BPs ternary complex to facilitate the degradation of IGF2BPs, but it also triggers cellular immune responses by activating retinoic acid-inducible gene-I (RIG-I), thereby regulating NSCLC progression (Li et al. 2021b).

To sum up, their study broadens the knowledge of m^6^A-modified circRNAs action in NSCLC progression, implying that circNDUFB2 may have immunotherapy potentials for NSCLC.

### Glioma

Glioma, an intracranial malignant tumor, has a high mortality and morbidity rate (Ostrom et al. 2014). Recent research into the molecular mechanism of glioma malignant proliferation has sparked widespread concern.

By using m^6^A level detection and MeRIP assays, Wu et al. discovered that METTL3-mediated m^6^A modification can enhance the stability and expression of circDLC1, thereby promoting the competitive binding of circDLC1 and miR-671-5p, facilitating Catenin Beta Interacting Protein 1 (CTNNBIP1) transcription, and ultimately suppressing the malignant proliferation of glioma cells (Wu et al. 2022).

This study first reported the mechanism of METTL3-mediated m^6^A modification of circDLC1 on the malignant proliferation of glioma cells, shedding light on glioma treatment.

### M^6^A-modified circRNAs and tumor chemoradiotherapy resistance

Increasing evidence suggests that m^6^A-modified circRNAs may also contribute to cancer chemotherapy resistance. For example, in sorafenib-resistant HCC cells, Xu et al. demonstrated that the m^6^A modification can increase its stability to regulate circRNA-SORE expression by using SRAMP, RMBase v2.0 database, and MeRIP assays, and that increased circRNA-SORE can sponge miR-103a-2-5p and miR-660-3p to activate Wingless-types/beta-catenin (Wnt/β-catenin) pathway and induce sorafenib resistance (Xu et al. 2020). Besides, the SRAMP database and MeRIP assays discovered that circMAP3K4 is highly enriched in the m^6^A modification, and further investigations revealed that IGF2BP1-mediated m^6^A recognition can translate circMAP3K4 into circMAP3K4 translation produced a 455 amino acid protein (circMAP3K4-455aa), thus preventing HCC cells from cisplatin-induced death (Duan et al. 2022). Additionally, recent research has explored how radiotherapy affects hypopharyngeal squamous cell carcinoma (HPSCC) prognosis. Diagnostics and treatments based on molecular biology are urgently needed to mitigate toxicity and adverse effects. For example, one study using MeRIP assays confirmed that METTL3 could stabilize the expression of circCUX1 through m^6^A modification in head and neck tumor cell lines. Notably, circCUX1 can bind to caspase 1 mRNA and inhibit its expression, thereby inhibiting caspase 1 mediated inflammation and developing tolerance to radiotherapy (Wu et al. 2021) (Table 3).

**Table 3:** Roles of m^6^A-modified circRNAs in various cancers

Cancer	CircRNA name	Regulation	M^6^A component	Fuction	Role in cancer	M^6^A identification methods and databases	Main mechanisms	References
CRC	circ3823	Up	ALKBH5/YTHDF2/YTHDF3	Eraser/reader/reader	Anti-oncogene	MeRIP assay, GEO and TCGA databases	Sponge miR-30c-5p to upregulate TCF7 expression	Guo et al. (2021)
CRC	circ1662	Up	METTL3	Writer	Oncogene	MeRIP assay	Bind to YAP1 protein and promote its nuclear transport to regulate SMAD3	Chen et al. (2021)
CRC	circNSUN2	Up	YTHDC1/IGF2BP2	Reader/reader	Oncogene	MeRIP assay	Bind to YTHDC1 and promote its export to the cytoplasm, as well as stabilize HMGA2 mRNA via circNSUN2-IGF2BP2-HMGA2 axis	Chen et al. (2019b)
PDGA	A series of circRNAs (circ0077837)	Up/down (down)	–	–	–	SRAMP, m^6^A RIP	-	Zhang et al. (2020)
GC	circORC5	Up	METTL14	Writer	Anti-oncogene	m^6^A-circRNA epitranscriptomic microarray, MeRIP assay	Sponge miR-30c-2-3p to regulate AKT1S1 and EIF4B expression	Fan et al. (2022)
HCC	circMAP2K4	Up	YTHDF1	Reader	Oncogene	TCGA and ICGC databases	Sponge hsa-miR-139-5p to regulate the expression of YTHDF1	Chi et al. 2021)
HCC	circDLC1	Down	KIAA1429	Writer	Oncogene	m^6^A-seq	Bind to HuR protein and block the interaction between HuR and MMP1 mRNAs	Liu et al. (2021)
HCC	circHPS5	Up	METTL3/YTHDC1	Writer/reader	Oncogene	MeRIP-seq, SRAMP, m^6^A RIP	Sponge miR-370 to regulate HMGA2 expression and expedite its cytoplasmic output	Rong et al. (2021)
BC	circMETTL3	Up	METTL3/METTL14/FTO	Writer/writer/eraser	Oncogene	circBank, m^6^A RIP	Sponge miR-31-5p to upregulate CKD1 expression	Li et al. 2021a)
CC	circE7	Up	METTL3	Writer	Oncogene	m^6^A RIP	Translate into E7 oncoprotein	Zhao et al. (2019)
CC	circARHGAP12	Up	IGF2BP2	Reader	Oncogene	MeRIP assay	Bind to IGF2BP2 to enhance FOXM1 mRNA stability	Ji et al. (2021)
NSCLC	circNDUFB2	Down	METTL3/METTL14/IGF2BPs	Writer/writer/reader	Oncogene	MeRIP assay	Act as a scaffold by forming a TRIM25/circNDUFB2/IGF2BPs ternary complex to facilitate the degradation of IGF2BPs and trigger cellular immune responses by activating RIG-I	Li et al. (2021b)
Glioma	circDLC1	Down	METTL3	Writer	Anti-oncogene	m^6^A level detection, MeRIP assay	Sponge miR-671-5p to facilitate the transcription of CTNNBIP1	Wu et al. (2022)
Sorafenib-resistant hepatocellular carcinoma	circRNA-SORE	Up	METTL3/METTL14/FTO	Writer/writer/eraser	Oncogene	SRAMP, RMBase v2.0, MeRIP assay	Sponge miR-103a-2-5p and miR-660-3p to activate Wnt/β-catenin pathway	Xu et al. (2020)
HCC	circMAP3K4	Up	IGF2BP1	Reader	Oncogene	SRAMP, MeRIP assay	Translate into circMAP3K4-455aa	Duan et al. (2022)
Radiotherapy-resistant hypopharyngeal squamous cell carcinoma	circCUX1	Up	METTL3	Writer	Oncogene	MeRIP assay	Bind to caspase 1 mRNA and inhibit its expression	Wu et al. (2021)

To sum up, those findings suggest that m^6^A-modified circRNAs may act as a potential therapeutic target for tumor chemotherapy and radiotherapy tolerance.

## Conclusion and remarks

Much evidence supports that epigenetic modification can affect RNAs involved in cellular processes. The m^6^A modification on circRNAs has been gradually identified and is also critical for human development and disease progression. Similar to the modification in mRNAs, the m^6^A modification in circRNAs can be written, removed, and read by the same regulators and perform specific biological functions. In terms of the biological function, m^6^A modification can regulate circRNA translation, nuclear-cytoplasmic transport, and degradation. Most importantly, m^6^A-modified circRNAs can participate in various physiological and pathological processes, particularly in cancers. That means m^6^A-modified circRNAs have a wide range of biological functions and a broad research space in the future.

Previous studies have shown that circRNAs are stable in blood and body fluids due to their unique structure of single-stranded, covalently closed circular transcripts, which can help them avoid exonuclease degradation. Hence, abnormal-expressed circRNAs in peripheral blood or body fluids have been proven useful as biomarkers for tumor diagnosis (Ge et al. 2022). One recent study has found that the m^6^A level in peripheral blood RNA combined with current tumor markers such as carcinoembryonic antigen (CEA) or m^6^A demethylases ALKBH5 and FTO can improve the diagnostic value of m^6^A, revealing that the m^6^A level in peripheral blood RNA can be a potential biomarker for GC diagnosis and follow-up (Ge et al. 2020). Additionally, several cancer treatments, including surgery, chemotherapy, radiotherapy, targeted therapy, and immunotherapy, have been widely applied over the past few decades, generally prolonging disease-free survival (PFS) and overall survival (OS) rates among cancer patients (Maji et al. 2018; Esfahani et al. 2020). However, due to the enormous tumor heterogeneity, cancer cells typically show primary or acquired drug resistance, leading to cancer treatment failure. For this reason, an increasing amount of research is focusing on less toxic therapies based on molecular biology. Aside from the m^6^A-modified circRNAs as therapeutic targets for tumor chemotherapy and radiotherapy resistance, the m^6^A regulators have also become therapeutic targets for tumors. For example, one research has revealed that ALKBH5-mediated alterations in m^6^A density can regulate the splicing and expression of mRNAs with potential roles in controlling tumor growth, thus suggesting that ALKBH5, the m^6^A demethylase, can be a potential therapeutic target for cancer treatment alone or in combination with immune checkpoint blockade (ICB) (Li et al. 2020). Nevertheless, more research is needed to comprehensively understanding how m^6^A regulatory factors function in cancer therapy. Furthermore, some methods for detecting m^6^A-modified circRNAs, such as dot blot, MeRIP assay, and MeRIP-seq, are widely used. Other methods, such as m^6^A-circRNA epitranscriptomic microarray, MazF PCR, and nanopore DRS, will require more proof-of-concept studies in the future.

Briefly, more studies on the biological functions and mechanisms of m^6^A-modified circRNAs are needed, especially in the following aspects: (i) Detecting whether the m^6^A level of m^6^A-modified circRNAs in peripheral blood or other liquid biopsy samples can serve as biomarkers or not; (ii) Determining how much m^6^A regulators and m^6^A-modified circRNAs play essential roles in cancer therapy and offer potential therapeutic targets; and (iii) Overcoming the technical obstacles and challenges in studying m^6^A-modified circRNAs. Based on previous research, we believe m^6^A-modified circRNAs will advance the field of the epigenome, provide novel potential targets for cancer progression, and generate more serendipity.

## Data Availability

Not applicable.

## Abbreviations

circRNAs: Circular RNAs
m^6^A: N^6^-methyladenosine
CVDs: Cardiovascular diseases
mRNAs: Messenger RNAs
ncRNAs: Non-coding RNAs
m^5^C: 5-Methylcytosines
5hmC: 5-Hydroxymethylcytosine
m^1^A: N^1^-methyladenosines
m^6^Am: N^6^, 2′-Odimethyladenosine
m^7^G: 7-Methylguanine
Ψ: Pseudouridine
CDS: Coding sequence
3′-UTRs: 3′-Untranslated regions
RBP: RNA binding protein
TSEN: TRNA splicing endonuclease
BHB: Bulge-helix-bulge motif
EcircRNAs: Exonic circRNAs
EIciRNAs: Exon–intron circRNAs
CiRNAs: Intronic circRNAs
TricRNAs: TRNA intronic circular RNAs
miRNA: MicroRNA
ceRNA: Competing endogenous RNA
IRES: Internal ribosome entry site
ITAFs: IRES-transacting factors
MTC, also named “writers”: Methyltransferases complex
METTL3: Methyltransferase-like 3 protein
METTL14: Methyltransferase-like 14 protein
WTAP: Wilms Tumor 1 Associated Protein
VIRMA, also called “Virilizer” or “KIAA1429”: Vir-like m^6^A methyltransferase associated
RBM15/15B: RNA recognition motif 15/15B
ZC3H13: Zinc finger CCCH domain-containing protein 13
CBLL1, also known as “HAKAI”: Cbl proto-oncogene-like 1
METTL16: Methyltransferase-like 16
METTL5: Methyltransferase-like 5
ZCCHC4: Zinc finger CCCH-Type containing 4
FTO, also known as “ALKBH9”: Fat mass and obesity-associated protein
ALKBH5: AlkB homolog 5
αKG: α-Ketoglutaric acid
YTH: YT521-B homology
YTHDF1: YTH domain family protein 1
YTHDF2: YTH domain family protein 2
YTHDF3: YTH domain family protein 3
YTHDC1: YTH domain containing 1
YTHDC2: YTH domain containing 2
CCR4-NOT: Carbon catabolite repressor 4-negative on TATA
P-body: Processing body
eIF3: Eukaryotic initiation factor 3
eIF4E: Eukaryotic initiation factor 4E
eIF4G: Eukaryotic initiation factor 4G
PABP: Poly(A) binding protein
HNRNPC: Heterogeneous nuclear ribonucleoprotein C1/C2
HNRNPG: Heterogeneous nuclear ribonucleoprotein G
HNRNPA2B1: Heterogeneous nuclear ribonucleoprotein A2B1
HNRNP: Heterogeneous nuclear ribonucleoprotein
IGF2BP1/2/3: Insulin-like growth factor 2 mRNA-binding protein 1/2/3
FMRP: Fragile X mental retardation protein
PRRC2A: Proline-rich spiral coil 2A
NGS: Next-generation sequencing
ELISA: Enzyme-linked immunosorbent assay
RNase R: Ribonuclease R
MeRIP: Methylated RNA immunoprecipitation
RIP: RNA immunoprecipitation
qPCR: Quantitative real-time polymerase chain reaction
MeRIP-seq: Methylated RNA immunoprecipitation and sequencing
nanopore DRS: Nanopore-based direct RNA sequencing
eIF4G2: Eukaryotic translation initiation factor 4 gamma 2
eIF3A: Eukaryotic initiation factor 3A
HPV: Human papillomavirus
Hel25E: Helicase at 25E
nuclear RNA helicase URH49: ATP-dependent RNA helicase DDX39A
dead box protein UAP56: Spliceosomal RNA helicase DDX39B
YAP1: Yes-associated protein 1
HMGA2: High Mobility Group AT-Hook 2
CDR1: Cerebellar degeneration associated protein 1
Ago2: Argonaute2
RNAi: RNA interference
HRSP12: Heat-responsive protein 12
RNase P: Ribonuclease P
MRP: Mitochondrial RNA processing
CRC: Colorectal carcinoma
GEO: Gene Expression Omnibus
TCGA: The Cancer Genome Atlas
TCF7: Transcription factor 7
EMT: Epithelial-mesenchymal transition
SMAD3: Mothers against decapentaplegic homolog 3
LM: Liver metastasis
RNA-EMSA: RNA electrophoretic mobility shift assay
FISH: Fluorescence in situ hybridization
GC: Gastric cancer
DECs: Differentiated expressed circRNAs
PDGA: Poorly differentiated gastric adenocarcinoma
AKT1S1: AKT1 substrate 1
EIF4B: Eukaryotic translation initiation factor 4B
HCC: Hepatocellular carcinoma
ICGC: International Cancer Genome Consortium
HuR: Human Antigen R
MMP1: Matrix metalloproteinase 1
BC: Breast cancer
CKD1: Cyclin-dependent kinases
CC: Cervical cancer
HPV: Human papillomavirus
FOXM1: Forkhead box M1
NSCLC: Non-small cell lung cancer
TRIM25: Tripartite motif containing 25
RIG-I: Retinoic acid-inducible gene-I
CTNNBIP1: Catenin Beta Interacting Protein 1
Wnt/β-catenin: Wingless-types/beta-catenin
circMAP3K4-455aa: CircMAP3K4 translation produced a 455 amino acid protein
HPSCC: Hypopharyngeal squamous cell carcinoma
CEA: Carcinoembryonic antigen
PFS: Disease-free survival rate
OS: Overall survival rate
ICB: Immune checkpoint blockade

## References

[CR1] Abakir A, Giles T, Cristini A, Foster J, Dai N, Starczak M, Rubio-Roldan A, Li M, Eleftheriou M, Crutchley J, Flatt L, Young L, Gaffney D, Denning C, Dalhus B, Emes R, Gackowski D, Corrêa I, Garcia-Perez J, Klungland A, Gromak N, Ruzov A (2020). *N*-methyladenosine regulates the stability of RNA:DNA hybrids in human cells. Nat Genet.

[CR2] Alarcón C, Goodarzi H, Lee H, Liu X, Tavazoie S, Tavazoie S (2015). HNRNPA2B1 is a mediator of m(6)A-dependent nuclear RNA processing events. Cell.

[CR3] Antanaviciute A, Baquero-Perez B, Watson C, Harrison S, Lascelles C, Crinnion L, Markham A, Bonthron D, Whitehouse A, Carr I (2017). Nm6aViewer: software for the detection, analysis, and visualization of *N*-methyladenosine peaks from mA-seq/ME-RIP sequencing data. RNA.

[CR4] Ashwal-Fluss R, Meyer M, Pamudurti N, Ivanov A, Bartok O, Hanan M, Evantal N, Memczak S, Rajewsky N, Kadener S (2014). circRNA biogenesis competes with pre-mRNA splicing. Mol Cell.

[CR5] Aufiero S, van den Hoogenhof M, Reckman Y, Beqqali A, van der Made I, Kluin J, Khan M, Pinto Y, Creemers E (2018). Cardiac circRNAs arise mainly from constitutive exons rather than alternatively spliced exons. RNA.

[CR6] Bawankar P, Lence T, Paolantoni C, Haussmann I, Kazlauskiene M, Jacob D, Heidelberger J, Richter F, Nallasivan M, Morin V, Kreim N, Beli P, Helm M, Jinek M, Soller M, Roignant J (2021). Hakai is required for stabilization of core components of the mA mRNA methylation machinery. Nat Commun.

[CR7] Boccaletto P, Stefaniak F, Ray A, Cappannini A, Mukherjee S, Purta E, Kurkowska M, Shirvanizadeh N, Destefanis E, Groza P, Avşar G, Romitelli A, Pir P, Dassi E, Conticello S, Aguilo F, Bujnicki J (2022). MODOMICS: a database of RNA modification pathways. Nucleic Acids Res.

[CR8] Broadbent K, Broadbent J, Ribacke U, Wirth D, Rinn J, Sabeti P (2015). Strand-specific RNA sequencing in *Plasmodium falciparum* malaria identifies developmentally regulated long non-coding RNA and circular RNA. BMC Genomics.

[CR9] Chen X, Yang T, Wang W, Xi W, Zhang T, Li Q, Yang A, Wang T (2019). Circular RNAs in immune responses and immune diseases. Theranostics.

[CR10] Chen R, Chen X, Xia L, Zhang J, Pan Z, Ma X, Han K, Chen J, Judde J, Deas O, Wang F, Ma N, Guan X, Yun J, Wang F, Xu R, Xie D (2019). *N*-methyladenosine modification of circNSUN2 facilitates cytoplasmic export and stabilizes HMGA2 to promote colorectal liver metastasis. Nat Commun.

[CR11] Chen C, Yuan W, Zhou Q, Shao B, Guo Y, Wang W, Yang S, Guo Y, Zhao L, Dang Q, Yang X, Wang G, Kang Q, Ji Z, Liu J, Sun Z (2021). N6-methyladenosine-induced circ1662 promotes metastasis of colorectal cancer by accelerating YAP1 nuclear localization. Theranostics.

[CR12] Chi F, Cao Y, Chen Y (2021). Analysis and validation of circRNA-miRNA network in regulating mA RNA methylation modulators reveals CircMAP2K4/miR-139–5p/YTHDF1 axis involving the proliferation of hepatocellular carcinoma. Front Oncol.

[CR13] Danan M, Schwartz S, Edelheit S, Sorek R (2012). Transcriptome-wide discovery of circular RNAs in Archaea. Nucleic Acids Res.

[CR14] Deng S, Zhang H, Zhu K, Li X, Ye Y, Li R, Liu X, Lin D, Zuo Z, Zheng J (2021). M6A2Target: a comprehensive database for targets of m6A writers, erasers and readers. Brief Bioinform.

[CR15] Di Timoteo G, Dattilo D, Centrón-Broco A, Colantoni A, Guarnacci M, Rossi F, Incarnato D, Oliviero S, Fatica A, Morlando M, Bozzoni I (2020). Modulation of circRNA metabolism by mA modification. Cell Rep.

[CR16] Dominissini D, Moshitch-Moshkovitz S, Schwartz S, Salmon-Divon M, Ungar L, Osenberg S, Cesarkas K, Jacob-Hirsch J, Amariglio N, Kupiec M, Sorek R, Rechavi G (2012). Topology of the human and mouse m6A RNA methylomes revealed by m6A-seq. Nature.

[CR17] Du H, Zhao Y, He J, Zhang Y, Xi H, Liu M, Ma J, Wu L (2016). YTHDF2 destabilizes m(6)A-containing RNA through direct recruitment of the CCR4-NOT deadenylase complex. Nat Commun.

[CR18] Duan J, Chen W, Xie J, Zhang M, Nie R, Liang H, Mei J, Han K, Xiang Z, Wang F, Teng K, Chen R, Deng M, Yin Y, Zhang N, Xie D, Cai M (2022). A novel peptide encoded by N6-methyladenosine modified circMAP3K4 prevents apoptosis in hepatocellular carcinoma. Mol Cancer.

[CR19] Enuka Y, Lauriola M, Feldman M, Sas-Chen A, Ulitsky I, Yarden Y (2016). Circular RNAs are long-lived and display only minimal early alterations in response to a growth factor. Nucleic Acids Res.

[CR20] Esfahani K, Roudaia L, Buhlaiga N, Del Rincon S, Papneja N, Miller W (2020). A review of cancer immunotherapy: from the past, to the present, to the future. Curr Oncol.

[CR21] Fan H, Chen Z, Chen X, Chen M, Yi Y, Zhu J, Zhang J (2022). METTL14-mediated mA modification of circORC5 suppresses gastric cancer progression by regulating miR-30c-2–3p/AKT1S1 axis. Mol Cancer.

[CR22] Ge L, Zhang N, Chen Z, Song J, Wu Y, Li Z, Chen F, Wu J, Li D, Li J, Wang C, Wang H, Wang J (2020). Level of N6-methyladenosine in peripheral blood RNA: a novel predictive biomarker for gastric cancer. Clin Chem.

[CR23] Ge L, Sun Y, Shi Y, Liu G, Teng F, Geng Z, Chen X, Xu H, Xu J, Jia X (2022). Plasma circRNA microarray profiling identifies novel circRNA biomarkers for the diagnosis of ovarian cancer. J Ovarian Res.

[CR24] Gomes C, Schroen B, Kuster G, Robinson E, Ford K, Squire I, Heymans S, Martelli F, Emanueli C, Devaux Y (2020). Regulatory RNAs in heart failure. Circulation.

[CR25] Guo J, Agarwal V, Guo H, Bartel D (2014). Expanded identification and characterization of mammalian circular RNAs. Genome Biol.

[CR26] Guo Y, Guo Y, Chen C, Fan D, Wu X, Zhao L, Shao B, Sun Z, Ji Z (2021). Circ3823 contributes to growth, metastasis and angiogenesis of colorectal cancer: involvement of miR-30c-5p/TCF7 axis. Mol Cancer.

[CR27] Hansen T, Wiklund E, Bramsen J, Villadsen S, Statham A, Clark S, Kjems J (2011). miRNA-dependent gene silencing involving Ago2-mediated cleavage of a circular antisense RNA. EMBO J.

[CR28] Hansen T, Jensen T, Clausen B, Bramsen J, Finsen B, Damgaard C, Kjems J (2013). Natural RNA circles function as efficient microRNA sponges. Nature.

[CR29] Hirsch F, Scagliotti G, Mulshine J, Kwon R, Curran W, Wu Y, Paz-Ares L (2017). Lung cancer: current therapies and new targeted treatments. Lancet.

[CR30] Hofheinz R, Stintzing S (2019). Study evidence confirms current clinical practice in refractory metastatic colorectal cancer: the ReDOS trial. Lancet Oncol.

[CR31] Howe K, Achuthan P, Allen J, Allen J, Alvarez-Jarreta J, Amode M, Armean I, Azov A, Bennett R, Bhai J, Billis K, Boddu S, Charkhchi M, Cummins C, Da Rin Fioretto L, Davidson C, Dodiya K, El Houdaigui B, Fatima R, Gall A, Garcia Giron C, Grego T, Guijarro-Clarke C, Haggerty L, Hemrom A, Hourlier T, Izuogu O, Juettemann T, Kaikala V, Kay M, Lavidas I, Le T, Lemos D, Gonzalez Martinez J, Marugán J, Maurel T, McMahon A, Mohanan S, Moore B, Muffato M, Oheh D, Paraschas D, Parker A, Parton A, Prosovetskaia I, Sakthivel M, Salam A, Schmitt B, Schuilenburg H, Sheppard D, Steed E, Szpak M, Szuba M, Taylor K, Thormann A, Threadgold G, Walts B, Winterbottom A, Chakiachvili M, Chaubal A, De Silva N, Flint B, Frankish A, Hunt S, Iisley G, Langridge N, Loveland J, Martin F, Mudge J, Morales J, Perry E, Ruffier M, Tate J, Thybert D, Trevanion S, Cunningham F, Yates A, Zerbino D, Flicek P (2021). Ensembl. Nucleic Acids Res.

[CR32] Hsu P, Shi H, Zhu A, Lu Z, Miller N, Edens B, Ma Y, He C (2019). NThe RNA-binding protein FMRP facilitates the nuclear export of -methyladenosine-containing mRNAs. J Biol Chem.

[CR33] Huang C, Liang D, Tatomer D, Wilusz J (2018). A length-dependent evolutionarily conserved pathway controls nuclear export of circular RNAs. Genes Dev.

[CR34] Huang Q, Guo H, Wang S, Ma Y, Chen H, Li H, Li J, Li X, Yang F, Qiu M, Zhao S, Wang J (2020). A novel circular RNA, circXPO1, promotes lung adenocarcinoma progression by interacting with IGF2BP1. Cell Death Dis.

[CR35] Huang W, Ling Y, Zhang S, Xia Q, Cao R, Fan X, Fang Z, Wang Z, Zhang G (2021). TransCirc: an interactive database for translatable circular RNAs based on multi-omics evidence. Nucleic Acids Res.

[CR36] Imanishi M, Tsuji S, Suda A, Futaki S (2017). Detection of *N*-methyladenosine based on the methyl-sensitivity of MazF RNA endonuclease. Chem Commun.

[CR37] Jan van Zonneveld A, Kölling M, Bijkerk R, Lorenzen J (2021). Circular RNAs in kidney disease and cancer. Nat Rev Nephrol.

[CR38] Jeck W, Sorrentino J, Wang K, Slevin M, Burd C, Liu J, Marzluff W, Sharpless N (2013). Circular RNAs are abundant, conserved, and associated with ALU repeats. RNA.

[CR39] Ji P, Wu W, Chen S, Zheng Y, Zhou L, Zhang J, Cheng H, Yan J, Zhang S, Yang P, Zhao F (2019). Expanded expression landscape and prioritization of circular RNAs in mammals. Cell Rep.

[CR40] Ji F, Lu Y, Chen S, Yu Y, Lin X, Zhu Y, Luo X (2021). IGF2BP2-modified circular RNA circARHGAP12 promotes cervical cancer progression by interacting mA/FOXM1 manner. Cell Death Discov.

[CR41] Jia G, Fu Y, Zhao X, Dai Q, Zheng G, Yang Y, Yi C, Lindahl T, Pan T, Yang Y, He C (2011). N6-methyladenosine in nuclear RNA is a major substrate of the obesity-associated FTO. Nat Chem Biol.

[CR42] Jia R, Xiao M, Li Z, Shan G, Huang C (2019). Defining an evolutionarily conserved role of GW182 in circular RNA degradation. Cell Discov.

[CR43] Jiang T, Xia Y, Lv J, Li B, Li Y, Wang S, Xuan Z, Xie L, Qiu S, He Z, Wang L, Xu Z (2021). A novel protein encoded by circMAPK1 inhibits progression of gastric cancer by suppressing activation of MAPK signaling. Mol Cancer.

[CR44] Kim G, Imam H, Siddiqui A (2021). The RNA binding proteins YTHDC1 and FMRP regulate the nuclear export of -methyladenosine-modified hepatitis B virus transcripts and affect the viral life cycle. J Virol.

[CR45] Knuckles P, Lence T, Haussmann I, Jacob D, Kreim N, Carl S, Masiello I, Hares T, Villaseñor R, Hess D, Andrade-Navarro M, Biggiogera M, Helm M, Soller M, Bühler M, Roignant J (2018). Zc3h13/Flacc is required for adenosine methylation by bridging the mRNA-binding factor Rbm15/Spenito to the mA machinery component Wtap/Fl(2)d. Genes Dev.

[CR46] Kristensen L, Andersen M, Stagsted L, Ebbesen K, Hansen T, Kjems J (2019). The biogenesis, biology and characterization of circular RNAs. Nat Rev Genet.

[CR47] Legnini I, Di Timoteo G, Rossi F, Morlando M, Briganti F, Sthandier O, Fatica A, Santini T, Andronache A, Wade M, Laneve P, Rajewsky N, Bozzoni I (2017). Circ-ZNF609 is a circular RNA that can be translated and functions in myogenesis. Mol Cell.

[CR48] Li Z, Huang C, Bao C, Chen L, Lin M, Wang X, Zhong G, Yu B, Hu W, Dai L, Zhu P, Chang Z, Wu Q, Zhao Y, Jia Y, Xu P, Liu H, Shan G (2015). Exon-intron circular RNAs regulate transcription in the nucleus. Nat Struct Mol Biol.

[CR49] Li N, Kang Y, Wang L, Huff S, Tang R, Hui H, Agrawal K, Gonzalez G, Wang Y, Patel S, Rana T (2020). ALKBH5 regulates anti-PD-1 therapy response by modulating lactate and suppressive immune cell accumulation in tumor microenvironment. Proc Natl Acad Sci USA.

[CR50] Li Z, Yang H, Dai X, Zhang X, Huang Y, Shi L, Wei J, Ding Q (2021). CircMETTL3, upregulated in a m6A-dependent manner, promotes breast cancer progression. Int J Biol Sci.

[CR51] Li B, Zhu L, Lu C, Wang C, Wang H, Jin H, Ma X, Cheng Z, Yu C, Wang S, Zuo Q, Zhou Y, Wang J, Yang C, Lv Y, Jiang L, Qin W (2021). circNDUFB2 inhibits non-small cell lung cancer progression via destabilizing IGF2BPs and activating anti-tumor immunity. Nat Commun.

[CR52] Liu N, Dai Q, Zheng G, He C, Parisien M, Pan T (2015). N(6)-methyladenosine-dependent RNA structural switches regulate RNA-protein interactions. Nature.

[CR53] Liu N, Zhou K, Parisien M, Dai Q, Diatchenko L, Pan T (2017). N6-methyladenosine alters RNA structure to regulate binding of a low-complexity protein. Nucleic Acids Res.

[CR54] Liu W, Yan J, Zhang Z, Pian H, Liu C, Li Z (2018). Identification of a selective DNA ligase for accurate recognition and ultrasensitive quantification of *N*-methyladenosine in RNA at one-nucleotide resolution. Chem Sci.

[CR55] Liu M, Wang Q, Shen J, Yang B, Ding X (2019). Circbank: a comprehensive database for circRNA with standard nomenclature. RNA Biol.

[CR56] Liu H, Lan T, Li H, Xu L, Chen X, Liao H, Chen X, Du J, Cai Y, Wang J, Li X, Huang J, Yuan K, Zeng Y (2021). Circular RNA circDLC1 inhibits MMP1-mediated liver cancer progression via interaction with HuR. Theranostics.

[CR57] Lu Z, Filonov G, Noto J, Schmidt C, Hatkevich T, Wen Y, Jaffrey S, Matera A (2015). Metazoan tRNA introns generate stable circular RNAs in vivo. RNA.

[CR58] Luo X, Li H, Liang J, Zhao Q, Xie Y, Ren J, Zuo Z (2021). RMVar: an updated database of functional variants involved in RNA modifications. Nucleic Acids Res.

[CR59] Ma H, Wang X, Cai J, Dai Q, Natchiar S, Lv R, Chen K, Lu Z, Chen H, Shi Y, Lan F, Fan J, Klaholz B, Pan T, Shi Y, He C (2019). *N*-Methyladenosine methyltransferase ZCCHC4 mediates ribosomal RNA methylation. Nat Chem Biol.

[CR60] Maji S, Panda S, Samal S, Shriwas O, Rath R, Pellecchia M, Emdad L, Das S, Fisher P, Dash R (2018). Bcl-2 antiapoptotic family proteins and chemoresistance in cancer. Adv Cancer Res.

[CR61] Mao Y, Dong L, Liu X, Guo J, Ma H, Shen B, Qian S (2019). mA in mRNA coding regions promotes translation via the RNA helicase-containing YTHDC2. Nat Commun.

[CR62] Memczak S, Jens M, Elefsinioti A, Torti F, Krueger J, Rybak A, Maier L, Mackowiak S, Gregersen L, Munschauer M, Loewer A, Ziebold U, Landthaler M, Kocks C, le Noble F, Rajewsky N (2013). Circular RNAs are a large class of animal RNAs with regulatory potency. Nature.

[CR63] Meyer K, Saletore Y, Zumbo P, Elemento O, Mason C, Jaffrey S (2012). Comprehensive analysis of mRNA methylation reveals enrichment in 3' UTRs and near stop codons. Cell.

[CR64] Meyer K, Patil D, Zhou J, Zinoviev A, Skabkin M, Elemento O, Pestova T, Qian S, Jaffrey S (2015). 5' UTR m(6)A promotes cap-independent translation. Cell.

[CR65] Nahand J, Jamshidi S, Hamblin M, Mahjoubin-Tehran M, Vosough M, Jamali M, Khatami A, Moghoofei M, Baghi H, Mirzaei H (2020). Circular RNAs: new epigenetic signatures in viral infections. Front Microbiol.

[CR66] Nombela P, Miguel-López B, Blanco S (2021). The role of mA, mC and Ψ RNA modifications in cancer: novel therapeutic opportunities. Mol Cancer.

[CR67] Ostrom Q, Bauchet L, Davis F, Deltour I, Fisher J, Langer C, Pekmezci M, Schwartzbaum J, Turner M, Walsh K, Wrensch M, Barnholtz-Sloan J (2014). The epidemiology of glioma in adults: a "state of the science" review. Neuro-Oncol.

[CR68] Park O, Ha H, Lee Y, Boo S, Kwon D, Song H, Kim Y (2019). Endoribonucleolytic cleavage of mA-containing RNAs by RNase P/MRP complex. Mol Cell.

[CR69] Patil D, Chen C, Pickering B, Chow A, Jackson C, Guttman M, Jaffrey S (2016). m(6)A RNA methylation promotes XIST-mediated transcriptional repression. Nature.

[CR70] Pendleton K, Chen B, Liu K, Hunter O, Xie Y, Tu B, Conrad N (2017). The U6 snRNA mA methyltransferase METTL16 Regulates SAM synthetase intron retention. Cell.

[CR71] Ping X, Sun B, Wang L, Xiao W, Yang X, Wang W, Adhikari S, Shi Y, Lv Y, Chen Y, Zhao X, Li A, Yang Y, Dahal U, Lou X, Liu X, Huang J, Yuan W, Zhu X, Cheng T, Zhao Y, Wang X, Rendtlew Danielsen J, Liu F, Yang Y (2014). Mammalian WTAP is a regulatory subunit of the RNA N6-methyladenosine methyltransferase. Cell Res.

[CR72] Pinto R, Vågbø C, Jakobsson M, Kim Y, Baltissen M, O'Donohue M, Guzmán U, Małecki J, Wu J, Kirpekar F, Olsen J, Gleizes P, Vermeulen M, Leidel S, Slupphaug G, Falnes P (2020). The human methyltransferase ZCCHC4 catalyses N6-methyladenosine modification of 28S ribosomal RNA. Nucleic Acids Res.

[CR73] Rong D, Wu F, Lu C, Sun G, Shi X, Chen X, Dai Y, Zhong W, Hao X, Zhou J, Xia Y, Tang W, Wang X (2021). m6A modification of circHPS5 and hepatocellular carcinoma progression through HMGA2 expression. Mol Ther Nucleic Acids.

[CR74] Rybak-Wolf A, Stottmeister C, Glažar P, Jens M, Pino N, Giusti S, Hanan M, Behm M, Bartok O, Ashwal-Fluss R, Herzog M, Schreyer L, Papavasileiou P, Ivanov A, Öhman M, Refojo D, Kadener S, Rajewsky N (2015). Circular RNAs in the mammalian brain are highly abundant, conserved, and dynamically expressed. Mol Cell.

[CR75] Salzman J, Gawad C, Wang P, Lacayo N, Brown P (2012). Circular RNAs are the predominant transcript isoform from hundreds of human genes in diverse cell types. PLoS ONE.

[CR76] Sanger H, Klotz G, Riesner D, Gross H, Kleinschmidt A (1976). Viroids are single-stranded covalently closed circular RNA molecules existing as highly base-paired rod-like structures. Proc Natl Acad Sci USA.

[CR77] Schmidt C, Giusto J, Bao A, Hopper A, Matera A (2019). Molecular determinants of metazoan tricRNA biogenesis. Nucleic Acids Res.

[CR78] Schwartz S, Mumbach M, Jovanovic M, Wang T, Maciag K, Bushkin G, Mertins P, Ter-Ovanesyan D, Habib N, Cacchiarelli D, Sanjana N, Freinkman E, Pacold M, Satija R, Mikkelsen T, Hacohen N, Zhang F, Carr S, Lander E, Regev A (2014). Perturbation of m6A writers reveals two distinct classes of mRNA methylation at internal and 5' sites. Cell Rep.

[CR79] Shang Q, Yang Z, Jia R, Ge S (2019). The novel roles of circRNAs in human cancer. Mol Cancer.

[CR80] Shi H, Wang X, Lu Z, Zhao B, Ma H, Hsu P, Liu C, He C (2017). YTHDF3 facilitates translation and decay of *N*-methyladenosine-modified RNA. Cell Res.

[CR81] Sung H, Ferlay J, Siegel R, Laversanne M, Soerjomataram I, Jemal A, Bray F (2021). Global cancer statistics 2020: GLOBOCAN estimates of incidence and mortality worldwide for 36 cancers in 185 countries. Cancer J Clin.

[CR82] Suzuki H, Zuo Y, Wang J, Zhang M, Malhotra A, Mayeda A (2006). Characterization of RNase R-digested cellular RNA source that consists of lariat and circular RNAs from pre-mRNA splicing. Nucleic Acids Res.

[CR83] van Tran N, Ernst F, Hawley B, Zorbas C, Ulryck N, Hackert P, Bohnsack K, Bohnsack M, Jaffrey S, Graille M, Lafontaine D (2019). The human 18S rRNA m6A methyltransferase METTL5 is stabilized by TRMT112. Nucleic Acids Res.

[CR84] Venø M, Hansen T, Venø S, Clausen B, Grebing M, Finsen B, Holm I, Kjems J (2015). Spatio-temporal regulation of circular RNA expression during porcine embryonic brain development. Genome Biol.

[CR85] Wang P, Bao Y, Yee M, Barrett S, Hogan G, Olsen M, Dinneny J, Brown P, Salzman J (2014). Circular RNA is expressed across the eukaryotic tree of life. PLoS ONE.

[CR86] Wang X, Zhao B, Roundtree I, Lu Z, Han D, Ma H, Weng X, Chen K, Shi H, He C (2015). N(6)-methyladenosine modulates messenger RNA translation efficiency. Cell.

[CR87] Wang P, Doxtader K, Nam Y (2016). structural basis for cooperative function of Mettl3 and Mettl14 methyltransferases. Mol Cell.

[CR88] Wang Y, Wang H, Xi F, Wang H, Han X, Wei W, Zhang H, Zhang Q, Zheng Y, Zhu Q, Kohnen M, Reddy A, Gu L (2020). Profiling of circular RNA *N*-methyladenosine in moso bamboo (*Phyllostachys edulis*) using nanopore-based direct RNA sequencing. J Integr Plant Biol.

[CR89] Widagdo J, Anggono V, Wong J (2022). The multifaceted effects of YTHDC1-mediated nuclear mA recognition. Trends Genet.

[CR90] Wojtas M, Pandey R, Mendel M, Homolka D, Sachidanandam R, Pillai R (2017). Regulation of mA transcripts by the 3'→5' RNA helicase YTHDC2 is essential for a successful meiotic program in the mammalian germline. Mol Cell.

[CR91] Wu N, Yuan Z, Du K, Fang L, Lyu J, Zhang C, He A, Eshaghi E, Zeng K, Ma J, Du W, Yang B (2019). Translation of yes-associated protein (YAP) was antagonized by its circular RNA via suppressing the assembly of the translation initiation machinery. Cell Death Differ.

[CR92] Wu R, Li A, Sun B, Sun J, Zhang J, Zhang T, Chen Y, Xiao Y, Gao Y, Zhang Q, Ma J, Yang X, Liao Y, Lai W, Qi X, Wang S, Shu Y, Wang H, Wang F, Yang Y, Yuan Z (2019). A novel mA reader Prrc2a controls oligodendroglial specification and myelination. Cell Res.

[CR93] Wu P, Fang X, Liu Y, Tang Y, Wang W, Li X, Fan Y (2021). N6-methyladenosine modification of circCUX1 confers radioresistance of hypopharyngeal squamous cell carcinoma through caspase1 pathway. Cell Death Dis.

[CR94] Wu Q, Yin X, Zhao W, Xu W, Chen L (2022). Molecular mechanism of mA methylation of circDLC1 mediated by RNA methyltransferase METTL3 in the malignant proliferation of glioma cells. Cell Death Discov.

[CR95] Xia X, Li X, Li F, Wu X, Zhang M, Zhou H, Huang N, Yang X, Xiao F, Liu D, Yang L, Zhang N (2019). A novel tumor suppressor protein encoded by circular AKT3 RNA inhibits glioblastoma tumorigenicity by competing with active phosphoinositide-dependent Kinase-1. Mol Cancer.

[CR96] Xu J, Wan Z, Tang M, Lin Z, Jiang S, Ji L, Gorshkov K, Mao Q, Xia S, Cen D, Zheng J, Liang X, Cai X (2020). *N*-methyladenosine-modified CircRNA-SORE sustains sorafenib resistance in hepatocellular carcinoma by regulating β-catenin signaling. Mol Cancer.

[CR97] Xuan J, Sun W, Lin P, Zhou K, Liu S, Zheng L, Qu L, Yang J (2018). RMBase v2.0: deciphering the map of RNA modifications from epitranscriptome sequencing data. Nucleic Acids Res.

[CR98] Yang Y, Fan X, Mao M, Song X, Wu P, Zhang Y, Jin Y, Yang Y, Chen L, Wang Y, Wong C, Xiao X, Wang Z (2017). Extensive translation of circular RNAs driven by *N*-methyladenosine. Cell Res.

[CR99] Ye Y, Feng W, Zhang J, Zhu K, Huang X, Pan L, Su J, Zheng Y, Li R, Deng S, Bai R, Zhuang L, Wei L, Deng J, Li M, Chen R, Lin D, Zuo Z, Zheng J (2021). Genome-wide identification and characterization of circular RNA mA modification in pancreatic cancer. Genome Med.

[CR100] You X, Vlatkovic I, Babic A, Will T, Epstein I, Tushev G, Akbalik G, Wang M, Glock C, Quedenau C, Wang X, Hou J, Liu H, Sun W, Sambandan S, Chen T, Schuman E, Chen W (2015). Neural circular RNAs are derived from synaptic genes and regulated by development and plasticity. Nat Neurosci.

[CR101] Zhang Y, Hamada M (2018). DeepM6ASeq: prediction and characterization of m6A-containing sequences using deep learning. BMC Bioinform.

[CR102] Zhang Y, Zhang X, Chen T, Xiang J, Yin Q, Xing Y, Zhu S, Yang L, Chen L (2013). Circular intronic long noncoding RNAs. Mol Cell.

[CR103] Zhang F, Kang Y, Wang M, Li Y, Xu T, Yang W, Song H, Wu H, Shu Q, Jin P (2018). Fragile X mental retardation protein modulates the stability of its m6A-marked messenger RNA targets. Hum Mol Genet.

[CR104] Zhang C, Wang J, Geng X, Tu J, Gao H, Li L, Zhou X, Wu H, Jing J, Pan W, Mou Y (2020). Circular RNA expression profile and m6A modification analysis in poorly differentiated adenocarcinoma of the stomach. Epigenomics.

[CR105] Zhao J, Lee E, Kim J, Yang R, Chamseddin B, Ni C, Gusho E, Xie Y, Chiang C, Buszczak M, Zhan X, Laimins L, Wang R (2019). Transforming activity of an oncoprotein-encoding circular RNA from human papillomavirus. Nat Commun.

[CR106] Zheng G, Dahl J, Niu Y, Fedorcsak P, Huang C, Li C, Vågbø C, Shi Y, Wang W, Song S, Lu Z, Bosmans R, Dai Q, Hao Y, Yang X, Zhao W, Tong W, Wang X, Bogdan F, Furu K, Fu Y, Jia G, Zhao X, Liu J, Krokan H, Klungland A, Yang Y, He C (2013). ALKBH5 is a mammalian RNA demethylase that impacts RNA metabolism and mouse fertility. Mol Cell.

[CR107] Zhou Y, Zeng P, Li Y, Zhang Z, Cui Q (2016). SRAMP: prediction of mammalian N6-methyladenosine (m6A) sites based on sequence-derived features. Nucleic Acids Res.

[CR108] Zhou C, Molinie B, Daneshvar K, Pondick J, Wang J, Van Wittenberghe N, Xing Y, Giallourakis C, Mullen A (2017). Genome-wide maps of m6A circRNAs identify widespread and cell-type-specific methylation patterns that are distinct from mRNAs. Cell Rep.

